# Allergic fungal rhinosinusitis (AFRS) - more than a fungal disease?

**DOI:** 10.1186/2045-7022-3-S2-P15

**Published:** 2013-07-16

**Authors:** Tineke Dutre, Surayie Al Dousary, Nan Zhang, Claus Bachert

**Affiliations:** 1 University Hospital Ghent, Ghent, Belgium; 2 Rhinology Research Chair, Medical College, King Saud University, Department of Otorhinolaryngology, Riyadh, Saudi Arabia; 3 Upper Airway Research Laboratory, University Ghent, Department of Otorhinolaryngology, Ghent, Belgium

## 

Allergic fungal rhinosinusitis (AFRS) is characterized by the growth of fungi, mostly Aspergillus sp., in the paranasal sinuses together with the formation of nasal polyps, peanut-butter like "allergic mucin" with fungal hyphae and typical CT-findings, as well as increased serum total IgE and Aspergillus-specific IgE concentrations. We here hypothesize that the increase in serum total IgE is caused by the local symbiosis of Asp. sp. with Staphylococcus aureus, a germ which is known for the production of enterotoxins with superantigenic properties. We demonstrate the presence of S. aureus specific IgE antibodies in the sera of AFRS patients, correlating with total serum IgE concentrations, as well as the coexistence of both, A. fumigatus and S. aureus, in biofilm-like formations on the sinus mucosa. Similar mechanisms and findings may apply for Allergic Broncho-Pulmonary Aspergillosis/Mykosis (ABPA/M). This knowledge may result in new diagnostic and therapeutic approaches including anti-IgE strategies.

